# Supplementary material for: In-Depth Analysis of the Pancreatic Extracellular Matrix during Development for Next-Generation Tissue Engineering
Source: Int J Mol Sci. 2023 Jun 17;24(12):10268. doi: 10.3390/ijms241210268 (PMC10299253; doi:10.3390/ijms241210268)
Supplement: Supplementary file 1 [file ijms-24-10268-s001.zip › Sup materials v2.pdf]

International Journal of
Molecular Sciences

MDPI

Supplementary Materials: The following supporting information can be downloaded at: www.mdpi.com/xxx/s1

Figure S1: Differential matrisome profile between E14.5 and P1 pancreatic tissue.

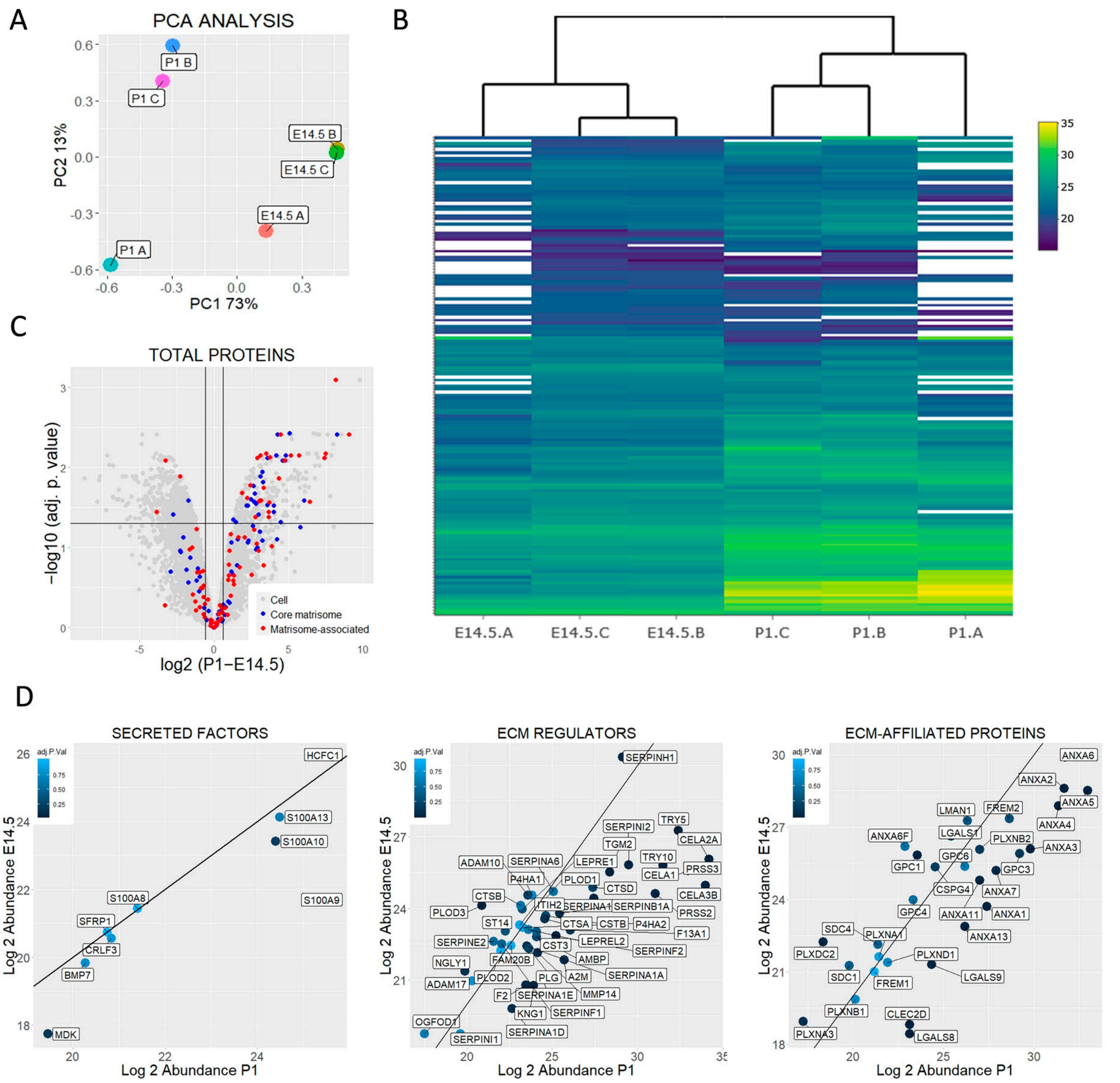

Figure S1. Differential matrisome profile between E14.5 and P1 pancreatic tissue. (A) Principal Component Analysis performed on all quantifiable proteins revealed separation of samples based on their developmental stage. **(B)** Heatmap of matrisome proteins across the 6 samples (3 at E145 and 3 at P1) showed more differences between the developmental groups than within the groups. Color indicates the abundance of the protein, from dark blue (low) to yellow (high). **(C)** Volcano plot of all quantifiable proteins based on the difference between P1 and E14.5. The proteins are color-coded by category: grey represents cellular proteins, blue represents core matrisome proteins, and red represents matrisome-associated proteins. **(D)** Scatter plots of the log2 abundance of matrisome-associated proteins in E14.5 pancreas (y-axis) versus abundance in P1 pancreas (x-axis). The left plot displays secreted factors, the middle plot displays ECM regulators, and the right plot displays ECM-affiliated proteins. The straight black line represents the median of the graph, with proteins above the line more detected in E14.5 pancreas and proteins below the line more detected in P1 pancreas. The colors indicate the adjusted p-value of the paired t-test performed between proteins of E14.5 and P1.

Figure S2: Viscoelasticity properties of pancreatic tissue and ECM during development.

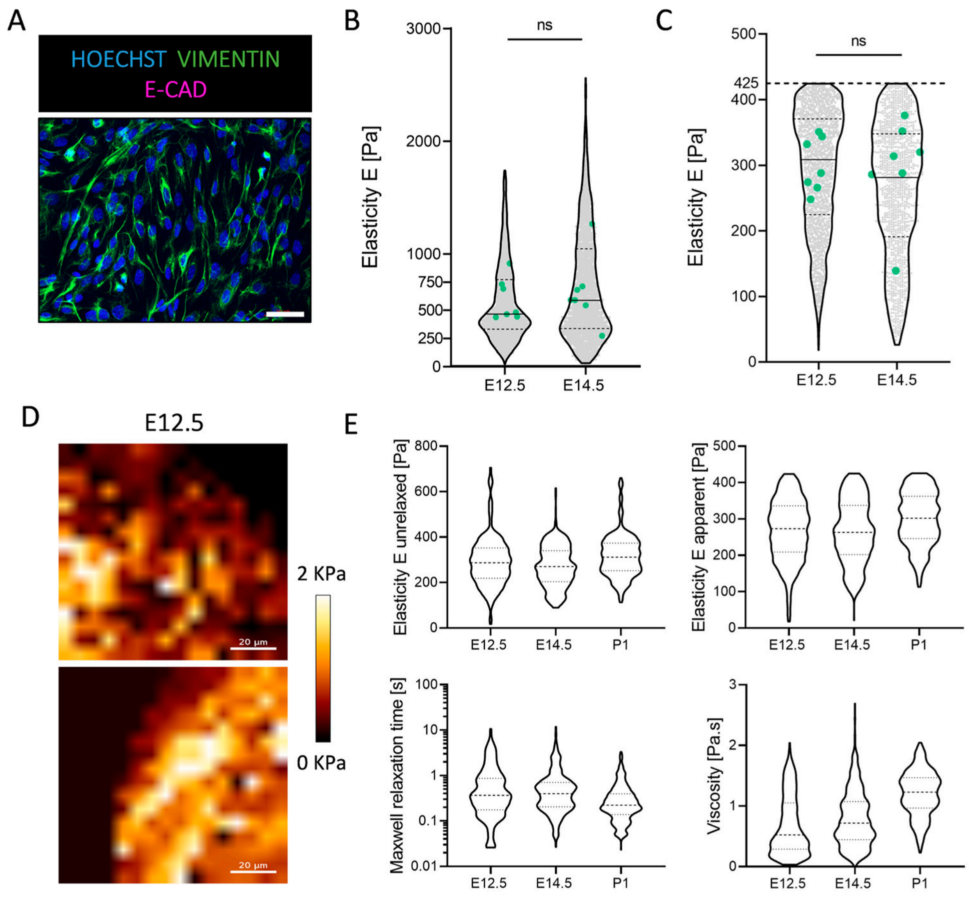

Figure S2. Differential matrisome profile between E14.5 and P1 pancreatic tissue. (A) Cultured P1 pancreatic cells immunolabelled with antibodies against E-CADHERIN (purple) and VIMENTIN (green), and counterstained with HOECHST (blue). Scale bar = 50 µm. **(B)** Global elasticity [Pa] of E12.5 and E14.5 pancreatic tissues. Grey points represent individual elasticity value computed from one force-displacement curve. Green points represent the mean of elasticity for one tissue sample (n=7 for E12.5 and E14.5). **(C)** Values within the range of cell's elasticity were excluded in E12.5 and E14.5 pancreas to display hypothetical ECM elasticity. Unpaired t-test performed on mean of biological replicates (green dots), ns = non significant. **(D)** Examples of elasticity maps at two distinct locations for E12.5 pancreatic tissue are displayed. The maps are obtained from an analyzed area of 100x100 µm with 32 acquisitions per side. Colors indicated the values of elasticity from low (0 KPa, dark) to high (1,5 or 2 KPa, bright). Scale bar = 20µm. **(E)** Violin plots of viscoelasticity parameters for E12.5 (n=4), E14.5 (n=7) and P1 (n=3) pancreatic ECM. The parameters include the unrelaxed and apparent elasticity [Pa], the Maxwell relaxation time [s] and the viscosity [Pa.s] which are determined by the fitting model developed by Abuhattum *et al.* [16] on AFM force-displacement curves with apparent elasticity below 425 Pa.

Figure S3: Efficiency of the decellularisation process and preservation of the 3D ECM structure.

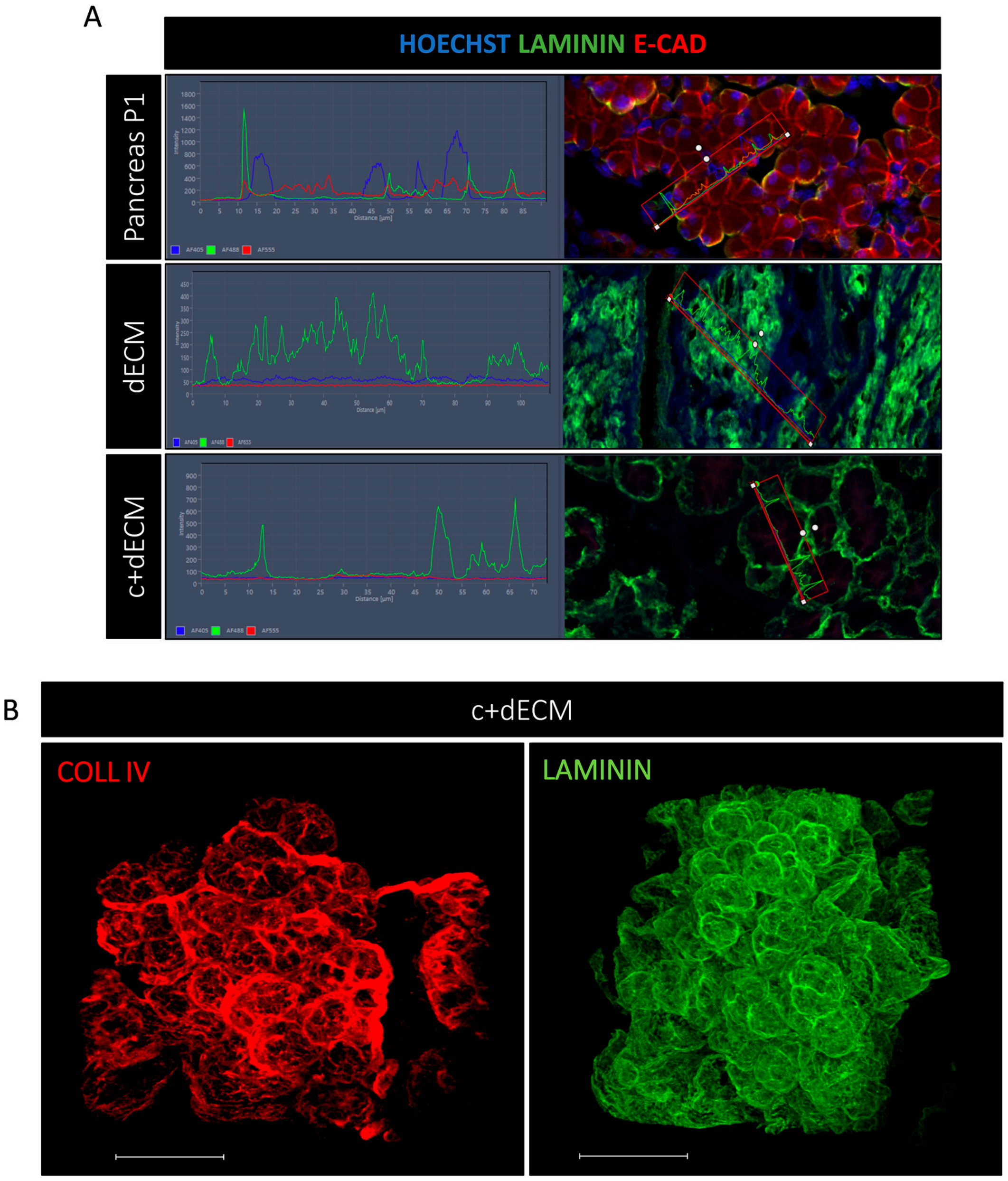

Figure S3: Efficiency of the decellularisation process and preservation of the 3D ECM structure.

(A) Immunolabelling of P1 pancreatic tissue, dECM and c+dECM scaffolds with antibodies directed against E-CADHERIN (red, AF633), LAMININ (green, AF488) and counterstained with HOECHST (blue, AF405). The intensity of immunofluorescence signals along the red line in fluorescent images (right) demonstrated the efficiency of the decellularization process; no HOECHST or E-CADHERIN are detected as compared to the native tissue. **(B)** c+dECM scaffolds labelled with antibodies directed against COLLAGEN IV (red) and LAMININ (green) and imaged with multi-photons microscopy. Images illustrate the preservation of basement membrane structure after the decellularization process. Scale = 60 µm.

Figure S4: Decellularization of E14.5 and P14 pancreatic tissues.

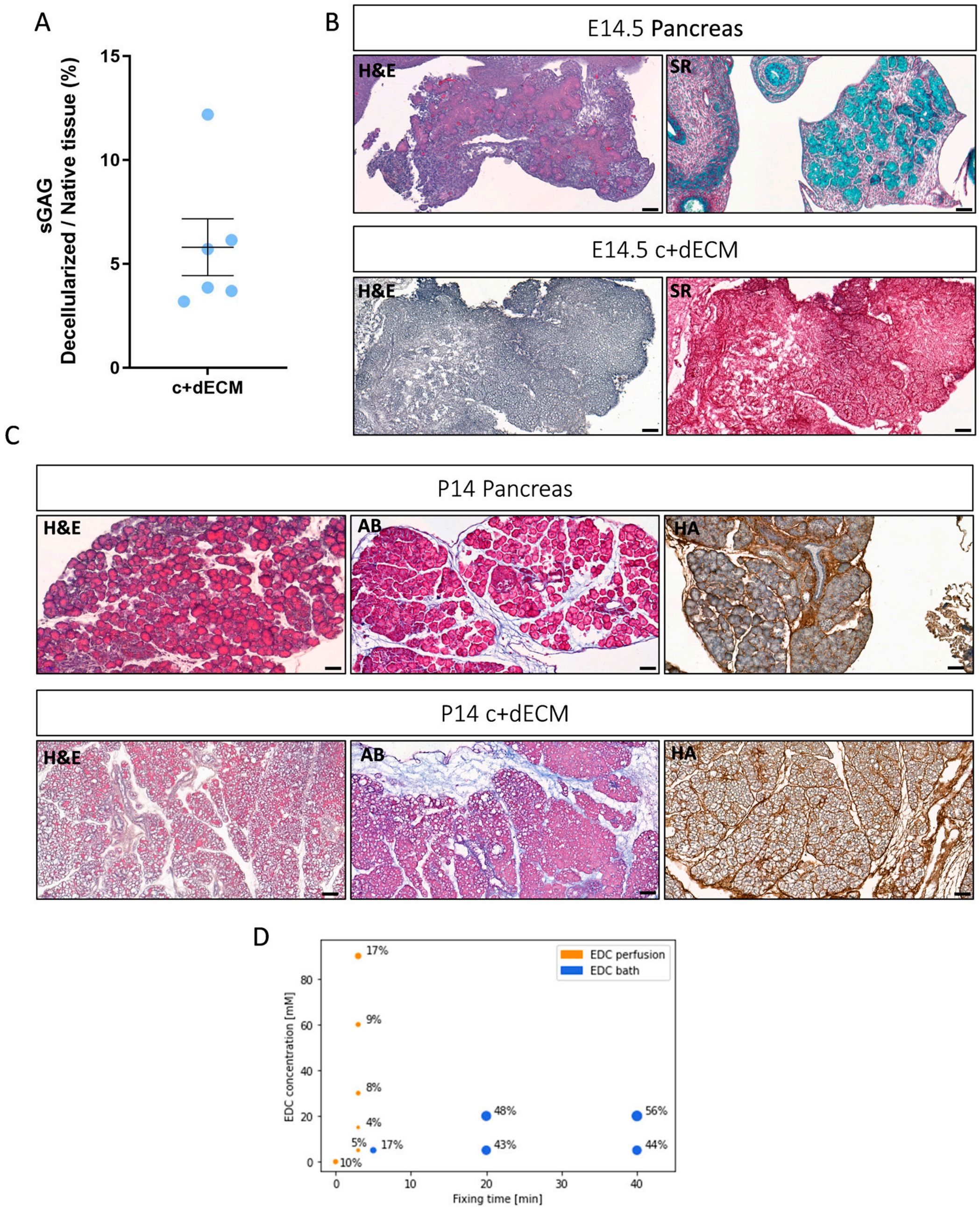

Figure S4: Decellularization of E14.5 and P14 pancreatic tissues.

(A) Quantification of sulfated GAG (sGAG) performed in P1 c+dECM scaffold reveals a 95% loss of GAG as compared to the native tissue (n=6). **(B)** Native (top) and decellularized E14.5 pancreatic tissue stained with H&E (at left) to assess removal of cellular components, and with Sirius red/fast green (at right) to detect collagenous protein (red) and cellular cytoplasm (green). Scale bars = 50 µm **(C)** Native (top) and decellularized (5 mM EDC for 20 min) P14 pancreatic tissue stained with H&E (left panels), Alcian blue (AB, middle panels), and immunolabeled for hyaluronic acid (HA, right panels) binding proteins. Scale bar = 50µm **(D)** Quantification of sGAG performed on P14 pancreatic tissue with various EDC concentration (y axis) and incubation time (x axis). Percentage represents the amount of sGAG quantified in the c+dECM scaffold divided by the amount of sGAG in native tissue. Colors correspond to the decellularization method. Orange: EDC was administrated by perfusion (injection in the left ventricle). Blue: dissected pancreas were immersed in EDC solution.

Figure S5: Biocompatibility of dECM and c+dECM scaffolds with pancreatic carcinoma cell line.

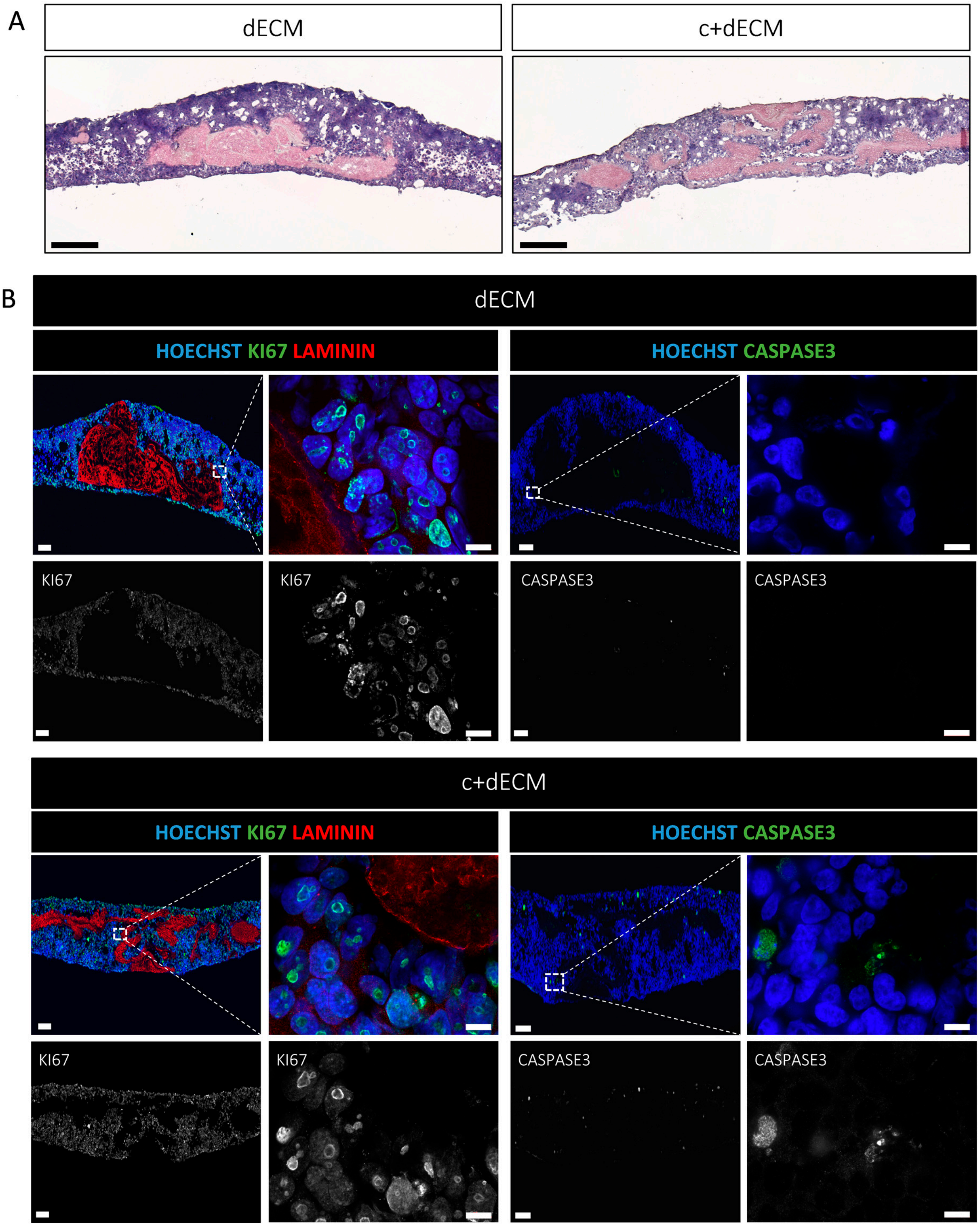

Figure S5: Biocompatibility of dECM and c+dECM scaffolds with pancreatic carcinoma cell line. dECM and c+dECM scaffolds on a semi-permeable filter were seeded with one million Panc-1 and cultured for 6 days. **(A)** H&E staining of the recellularized ECM scaffolds showed the aggregation of cells around both scaffolds. A better penetration of cells was observed in c+dECM scaffolds. **(B)** Immunolabelling of the re-cellularized ECM scaffolds with antibodies directed against Ki67 (green) or Caspase 3 (green), E-cadherin (purple), and Laminin (red), and counterstained with DAPI (blue), demonstrated the biocompatibility of both decellularization processes. Each panel includes an image of the entire structure (left) and a zoomed-in image (white square) for cellular resolution (right). The bottom panels show Ki67 or Caspase3 labeling

alone (white) from the corresponding top images. Scale bars, 200 μm (A), 100 μm (B, at left) and 10 μm (B, at right).
